# Lighting the Path: Advancing Research in Electrical Storm

**DOI:** 10.1016/j.jacadv.2025.102332

**Published:** 2025-11-07

**Authors:** Pouya Motazedian, F. Daniel Ramirez, Pietro Di Santo, Graeme Prosperi-Porta, David Nelson, Jason K. Lee, Richard Jung, Rebecca Mathew, George A. Wells, Benjamin Hibbert

**Affiliations:** a CAPITAL Research Group, Division of Cardiology, University of Ottawa Heart Institute, Ottawa, Ontario, Canada; b Division of Cardiology, University of Ottawa Heart Institute, Ottawa, Ontario, Canada; c School of Epidemiology and Public Health, University of Ottawa, Ottawa, Ontario, Canada; d Department of Cardiovascular Medicine, Mayo Clinic, Rochester, Minnesota, USA

**Keywords:** electrical storm, ventricular arrhythmia, evidence-based medicine, dexmedetomidine, stellate ganglion blockade, ventricular tachycardia

Ventricular electrical storm (ES) is a state of densely clustered ventricular arrhythmias (VAs) that is associated with significant morbidity and mortality. It has become increasingly prevalent in cardiac and medical intensive care units, prompting a surge of contemporary reviews and new guidelines. Despite this growing attention, evidence-based recommendations for ES remain limited and are based almost exclusively on small observational studies.^1^ There is a pressing need to develop high-quality evidence-based definitions and data to advance the care of these patients.

## Challenges in defining the patient population

Recurrent VAs are associated with poor cardiovascular outcomes and increased mortality, with increased event frequency and density associated with greater risk.^2^ ES, being the most severe form of clustered VAs, is a life-threatening condition often requiring resource-intensive advanced inpatient therapies.^3^ Current guidelines define ES based on an arrhythmia criterion of three or more treated or sustained VAs in a 24-hour period.^3^^,^^4^ This threshold is somewhat arbitrary, stemming more from historical convention rather than from evidence showing a clear inflection point in risk at this threshold. Furthermore, while some studies have quantified long-term outcomes based on event frequency and density of VAs, no studies have identified a specific threshold for those at the highest risk of acute deterioration, which is the key component differentiating ES from clustered VAs.^2^, ^3^, ^4^

There are also inconsistencies in how VA events are defined when characterizing ES. European guidelines require that events should be separated by more than 5 minutes to be considered distinct, whereas this criterion is absent from the American guidelines.^3^^,^^4^ Although there may be a pathophysiologic rationale to use a time-based interval requirement to categorize densely recurring VAs as a single incessant event rather than ES, there is no compelling evidence that this distinction identifies patient populations with differing risk profiles or therapeutic responses.

Inconsistencies in diagnostic criteria have led to clinical studies with heterogeneous patient populations. Early ES trials from the 1990s defined ES as two or more hemodynamically VAs within a 24-hour period refractory to initial antiarrhythmic drug therapy. Although many of these patients would not meet current ES criteria, they represented a high-risk group as most had recurrent VAs and a 48-hour mortality of approximately a 20%.^5^, ^6^, ^7^ Meanwhile, in the only contemporary ES trial that demonstrated benefit of propranolol over metoprolol for the acute treatment of ES, no deaths occurred during the 3-month follow-up period.^8^ While the earlier studies were conducted before the widespread use of implantable cardioverter-defibrillators, they more accurately reflect the clinical severity that is inferred when describing ES. This raises concerns about whether the benefits observed in contemporary trials are generalizable across the full spectrum of ES patients.

These discrepancies also underscore the limitations of relying solely on arrhythmic criteria for ES diagnosis. Several factors likely influence the risk of refractory ES and patient outcomes, including prior arrhythmic history, underlying cardiac disease, antiarrhythmic drug use, and previous ablation procedures.^3^^,^^9^ These variables may alter the electrical threshold at which a patient’s condition becomes life-threatening. Moreover, the heterogeneity extends beyond diagnostic criteria, as there is currently no classification system based on underlying substrate, presence of triggers, or the characteristics of the presenting VAs.

The current landscape of ES research, marked by inconsistent definitions that are not supported by evidence, has resulted in challenges in appropriately diagnosing, risk-stratifying, and managing these patients. Relying on the current “one-size-fits-all” definition not only hampers the identification of higher-risk patients but also attempts to incorrectly combine a markedly heterogeneous patient population. A more comprehensive and standardized definition is therefore required to accurately characterize the ES patient population.

## Current evidence-based therapies in ES

### Quality of the literature

Despite the growing interest in ES, the majority of studies remain observational, descriptive, and lack control groups or comparators.^1^ Additionally, sample sizes are generally small, with a noticeable trend toward even smaller cohorts over time.^1^ Although such studies are valuable for the initial evaluation of ES therapies, significant publication bias limits their ability to support definitive conclusions regarding therapeutic efficacy. Given the current landscape, these lower-quality studies often influence practice disproportionately, despite their methodological limitations.

### Current evidence-based therapies

The current evidence-based categories of therapy for patients with ES consist of medical and supportive therapy, sympathetic blockade, and substrate modification.

To date, 4 clinical randomized controlled trials have evaluated the efficacy of medical therapy for ES. Three of these, conducted in the 1990s, compared bretylium and various amiodarone dosing protocols.^5^, ^6^, ^7^ While these remain the largest trials, their clinical relevance is limited due to several factors including the contemporary use of implantable cardioverter-defibrillators in the contemporary ES population, the adoption of significantly higher amiodarone loading strategies than those studied, and the lack of availability of bretylium in clinical practice. The most recent randomized controlled trials from 2018 was a single-center study involving only 60 patients that showed a reduction of VAs when using propranolol instead of metoprolol in addition to intravenous amiodarone for the initial management of hemodynamically stable ES. Given the absence of mortality in trial, this suggests a lower-risk ES population was recruited.^8^ Beyond the use of amiodarone and propranolol, there is limited evidence to guide medical therapy in this population.

Sympathetic blockade, aimed at reducing the catecholamine surge during ES, has only been studied in observational settings. In a multicenter data set from France, deep sedation with mechanical ventilation was associated with ES termination in 47% of patients but was associated with significant ventilator-related complications and mortality and requires the involvement of intensive care specialists.^10^ Although it is typically reserved for refractory cases, there is also considerable variability in the timing of this as a therapeutic intervention.^1^^,^^3^

More advanced interventions, such as sympathetic blockade by stellate ganglion block (SGB), require specialized expertise and resources that are currently available in only a minority of centers. SGB involves a percutaneous injection of an local anesthetic agent, typically ropivacaine, lidocaine, or bupivacaine, into the stellate ganglion in the neck to reduce sympathetic tone and reduce further VAs.^9^ In a recent systematic review, SGB was associated with a significant decrease in VA events before and after blockade, with complete resolution occurring in 70% of cases. While the evidence is encouraging, there are clear limitations in the evidence which have limited its widespread use. All studies so far are observational, lack comparator arms, and rely on before-and-after analyses, which are significantly confounded by the initiation of concurrent therapies and device setting changes.^9^ Moreover, many patients in these studies had SGB completed using an anatomical (ie “blind”) approach, whereas in pain clinics where SGB is used more frequently, ultrasound-guided SGB is the standard of care. Given the unclear efficacy of blind procedures and absence of robust study design and methodology, the current evidence supporting the use of SGB in ES is weak.

### Novel therapies and upcoming studies

Two active clinical trials are investigating treatments for the acute management of ES. The GANGSTER (GANGlion Stellate Block for Treatment of Electric StoRm) study is an 80-patient, sham-controlled, randomized trial evaluating the impact of SGB in patients with refractory ES (NCT05078684). The second trial, SEDATE (Study Evaluating Dexmedetomidine in the Acute Treatment of Electrical Storm), is a 192-patient, double-blind, placebo-controlled, randomized trial assessing the efficacy of dexmedetomidine, a commonly used sedative in intensive care units that does not require ventilatory support, for the initial management of ES (NCT06281977). Once completed, these trials are expected to provide more robust data on the acute antiarrhythmic effects of sympathetic blockade in ES.

## The need for evidence

Designing and conducting clinical studies in ES presents significant challenges, which have contributed to the substantial knowledge gap for a life-threatening condition commonly encountered in intensive care units. These difficulties stem not only from the challenges in clearly defining the patient population but also from the inherent complexities of conducting trials in critically ill cardiac patients. Despite these obstacles, progress is achievable. First, there is a historical precedent: large clinical trials involving this population were successfully conducted in the 1990s. Second, similar challenges have been addressed in the field of cardiogenic shock (CS), offering a valuable framework.

The recent advancements in CS, including the development of a practical classification model and the establishment of large granular and international data sets should serve as the next steps for ES. There should also be a focus on building international networks to enable high-quality clinical trials with the same methodological rigor seen in CS. The emergence of cardiac critical care as a recognized subspecialty should further facilitate this progress. As such, collaboration between the electrophysiology and cardiac critical care communities will be essential to drive research forward.

There is precedent, expertise, and a successful framework in place; what is now needed is focused attention and coordinated effort to advance the care of patients with ES.

## Funding support and author disclosures

Dr Hibbert has served as a consultant for Abbott and Edwards Lifesciences. All other authors have reported that they have no relationships relevant to the contents of this paper to disclose.
